# Effects of Exercise Intensity on Postexercise Endothelial Function and Oxidative Stress

**DOI:** 10.1155/2015/723679

**Published:** 2015-10-25

**Authors:** Conor McClean, Ryan A. Harris, Malcolm Brown, John C. Brown, Gareth W. Davison

**Affiliations:** ^1^Sport and Exercise Sciences Research Institute, Ulster University, Jordanstown, County Antrim BT37 0QB, UK; ^2^Division of Clinical & Translational Sciences, Georgia Prevention Center, Georgia Regents University, 1120 15th Street HS-1707, Augusta, GA 30912, USA

## Abstract

*Purpose*. To measure endothelial function and oxidative stress immediately, 90 minutes, and three hours after exercise of varying intensities.* Methods*. Sixteen apparently healthy men completed three exercise bouts of treadmill running for 30 minutes at 55% $\dot{V}O_{2max}$ (mild); 20 minutes at 75% $\dot{V}O_{2max}$ (moderate); or 5 minutes at 100% $\dot{V}O_{2max}$ (maximal) in random order. Brachial artery flow-mediated dilation (FMD) was assessed with venous blood samples drawn for measurement of endothelin-1 (ET-1), lipid hydroperoxides (LOOHs), and lipid soluble antioxidants.* Results*. LOOH increased immediately following moderate exercise (*P* < 0.05). ET-1 was higher immediately after exercise and 3 hours after exercise in the mild trial compared to maximal one (*P* < 0.05). Transient decreases were detected for ΔFMD/Shear_AUC_ from baseline following maximal exercise, but it normalised at 3 hours after exercise (*P* < 0.05). Shear rate was higher immediately after exercise in the maximal trial compared to mild exercise (*P* < 0.05). No changes in baseline diameter, peak diameter, absolute change in diameter, or FMD were observed following any of the exercise trials (*P* > 0.05).* Conclusions*. Acute exercise at different intensities elicits varied effects on oxidative stress, shear rate, and ET-1 that do not appear to mediate changes in endothelial function measured by FMD.

## 1. Introduction

It is well established that aerobic exercise training can improve endothelial function and flow-mediated dilation (FMD) responses [1], particularly in those with cardiovascular disease and related risk factors [2]. Despite this link, the acute effects of exercise on endothelial function and health have received relatively little attention [3] especially throughout the postexercise period (>1 hour after exercise) and in relation to oxidative stress, endothelin-1 (ET-1) production, or indeed exercise intensity. For instance, reductions in circulating ET-1, the potent vasoconstrictor, have been observed following aerobic exercise training [4], whereas elevated levels have been reported following acute intense exercise [5]. Moreover, acute strenuous aerobic exercise has been associated with transient reductions of FMD whereas moderate-intensity exercise is thought to confer beneficial effects, attributable to elevated reactive oxygen species (ROS) production at higher exercise intensities [6, 7]. An accumulation of free radical species can disrupt nitric oxide (NO) bioavailability and compromise endothelial-dependent vasodilation predisposing to vascular dysfunction [8]. Equally, it is recognised that free radicals/ROS are critical for cell function and in the regulation of intracellular signalling and gene expression [9]. Mechanistic scrutiny of such markers, deeper in the recovery period, is lacking and may be of valuable use to exercise and health professionals, especially when comparing various exercise doses, trying to delineate appropriate recommendations for exercise and physical activity guidelines for public health. Recently, there has been research interest in the potential of low volume, high intensity exercise to appeal to those who may struggle to participate in traditional endurance regimes, often aligned to the existing physical activity guidelines. Despite this, much of this research though has focused exclusively on high intensity interval training, using cycle ergometers. Scarce research exists investigating the effects of high intensity continuous running exercise for short durations. Therefore, the aim of this study was to investigate peripheral endothelial function and oxidative stress indices following acute aerobic exercise of differing intensities, not matched for volume, throughout the postexercise period. We hypothesize that differential responses to endothelial function and oxidative stress production will be observed throughout the postexercise period based on exercise intensity, whereby higher exercise intensities will cause an elevation in oxidative stress and ET-1 and a decrease in FMD.

## 2. Methods

### 2.1. Participants

Following the approval from a local research ethics committee and in accordance with the Declaration of Helsinki (1964), sixteen (*n* = 16) apparently healthy White Irish males (age: 27.2 ± 5.4 yrs; height; 173.0 ± 6.8 cm; body mass: 78.5 ± 18.0 kg; BMI: 25.3 ± 5.5 kg/m^2^; $\dot{V}O_{2max}$: 50.6 ± 6.5 mL·kg^−1^·min^−1^) volunteered to take part in the study. All participants were recreationally active, nonsmokers, and normotensive and were not taking medication or supplements, such as nonsteroidal anti-inflammatories, lipid lowering agents, and antioxidant supplements, which may have interfered with the relevant blood biochemistry. Before commencing the study, all participants completed written informed consent and a health screening questionnaire.

### 2.2. Experimental Design

Volunteers participated in a randomised controlled, crossover design, which involved four separate days of testing. Each bout of exercise was separated by at least four days and during this time participants were asked to maintain their normal dietary and physical activity behaviour. On the first day, anthropometric measures were taken and a maximal oxygen uptake ($\dot{V}O_{2max}$) test was performed to assess aerobic capacity. On the following three visits, participants completed three randomly allocated exercise trials which involved treadmill running for 30 minutes at 55%  $\dot{V}O_{2max}$ (mild); 20 minutes at 75%  $\dot{V}O_{2max}$ (moderate); or 5 minutes at 100%   $\dot{V}O_{2max}$ (maximal). Testing was completed in the morning after a standardized overnight fast (10 hours). Participants were also asked to refrain from drinking beverages containing caffeine. In the 48 hours before all laboratory visits, participants were required to abstain from alcohol and exercise, and in the three days prior to testing they were instructed to eat a similar diet. Experimental measures were taken at baseline, immediately following exercise (after), 90 minutes after exercise, and 3 hours after exercise. Prior to each exercise bout, a standardised warm-up was completed consisting of a 10-minute light jog (self-selected pace) on the treadmill followed by appropriate stretching and mobilisation exercises.

### 2.3. Anthropometric Measures

Height (to the nearest 0.1 cm) and body mass (to the nearest 0.1 kg) were measured using a freestanding stadiometer (Holtain Limited, Crymych, Dyfed, Britain) and digital scales (Seca, Vogel & Halke, Hamburg, Germany), respectively. This data was also used to calculate body mass index (BMI).

### 2.4. Maximal Incremental Exercise Test

Participants completed an incremental exercise protocol to volitional exhaustion on a motorised treadmill (Cosmos Lazer, Nussdorf, Germany). The test began at a fixed treadmill gradient of 0%, but the starting speed was self-selected by each participant. Each increment lasted for 1 minute and the incline of the treadmill was increased by 1%. Prior to the test, participants completed a 10-minute warm-up, during which the initial treadmill speed was established at 65% of predicted maximum heart rate (maximum heart rate: 208 – (0.7 × age)). Following daily calibration, participants were linked to an online computerised gas analysis system (Cosmed, Quark CPET, Italy), heart rate measurements were recorded every minute (Polar RS400, Finland), and vocal encouragement was given throughout the test. Criteria for assessing VO_2max_ included: an increase in oxygen consumption less than 2 mL·kg^−1^·min^−1^ in the last 2 minutes of exercise; a heart rate of within 5 beats·min^−1^ of predicted maximum; a respiratory exchange ratio exceeding 1.15. The rate of perceived exertion (RPE) was also measured using the Borg Scale [10].

### 2.5. Endothelial Function Measures

#### 2.5.1. Brachial Artery FMD

Briefly, upon arrival to the laboratory, participants rested in a supine position for 20 minutes before resting brachial artery FMD was measured using a 12 MHz linear transducer connected to a LOGIQ e ultrasound imaging device (GE Healthcare, UK). ECG gating (AccuSync 72, USA; GE Medical Systems, China) was used to ensure measurements taken at the same point in the cardiac cycle (end diastole). Baseline images were recorded for 10 cardiac cycles; frame collection was triggered via the ECG machine at the beginning of every R wave. The occlusion cuff was then rapidly inflated (E20 Rapid Cuff Inflator, AG101 Cuff Inflator Air Source, USA) to 250 mmHg for 5 minutes. Postcuff measurements were initiated ≥10 seconds before the cuff pressure was released and measurements were recorded for ≥2 minutes after release. Images were acquired using Vascular Imager software (Version 6.0.3, Medical Imaging Applications, USA). Arterial diameter was determined using offline edge detection software (Brachial Analyser for Research Version 5.7.0, Medical Imaging Applications, USA). FMD was performed and calculated according to the recent tutorial [11]. FMD was normalised to shear (shear area under the curve; Shear_AUC_) to control for the large intersubject variability in reactive hyperaemia-induced shear stress according to the methods of Padilla et al. [12]. Following completion of the exercise trial, FMD measurements were repeated immediately after exercise and 90 minutes and 3 hours after exercise. ΔFMD/Shear from baseline was calculated for each participant as the difference in FMD/Shear at each postexercise measurement point in relation to FMD/Shear at baseline for each respective trial day and then averaged.

#### 2.5.2. Blood Pressure

Systemic arterial blood pressure was measured with an automated Omron digital blood pressure monitor (Omron Healthcare, Japan). Participants were asked to rest in a supine position while measurements were taken from the dominant arm. Measurements were taken three times and a mean was calculated. Blood pressure readings were taken at rest, immediately after exercise, 90 minutes after exercise, and 3 hours after exercise.

### 2.6. Blood Biochemistry

#### 2.6.1. Blood Sampling

Blood samples were taken before exercise, immediately after exercise, and 3 hours after exercise from a prominent forearm vein while participants rested in a supine position. Immediately after collection, serum separating clot activator tubes (SST) were left for 15 minutes to clot at room temperature, while lithium heparinised tubes and K_3_ EDTA tubes were placed on ice. All blood tubes were centrifuged at 3500 rpm for 10 minutes, serum and plasma were removed, and aliquots were stored in 1.5 mL vials at −80°C for biochemical analysis. Postexercise packed cell volume and haemoglobin concentration were measured on whole blood to correct for acute exercise-induced plasma volume shifts using the equations of Dill and Costill [13].

#### 2.6.2. Measurement of ET-1

Serum was assayed for ET-1 using an immunoassay kit (Quantikine, R&D Systems, UK) which employed an ET-1 specific monoclonal antibody and an enzyme-linked monoclonal antibody to sandwich the ET-1 present in the sample wells. After preparation of reagents and standards, 150 *μ*L of Assay Diluent RD1-105 and 75 *μ*L of either the standard or sample were added to each well. This was then incubated at room temperature on a microplate shaker for one hour. The wells were washed four times by adding 400 *μ*L of wash buffer to each well. Once the wash buffer was completely removed, 200 *μ*L of ET-1 conjugate was added into each well and incubated for 3 hours at room temperature on a shaker. The wash step was repeated and the wells were incubated at room temperature for a further 30 minutes before 50 *μ*L of Stop Solution was added to each well. The optical density was read at 450 nm using a microplate reader (EL808 BioTek Instruments, USA).

#### 2.6.3. Measurement of Lipid Hydroperoxides

Serum lipid hydroperoxides (LOOHs) were measured using the ferrous iron/xylenol orange (FOX) assay [14]. Sample absorbance was measured using a UV spectrophotometer (UV mini-1240 Shimadzu, Mason Technologies, Ireland) at 560 nm. Intra- and interassay CV at 0.57 *μ*mol·L^−1^ = 4.6% and 6.0%, respectively.

#### 2.6.4. Lipid Soluble Antioxidants

Lipid soluble antioxidants were measured using the simultaneous high pressure liquid chromatography (HPLC) assay of Thurnham et al. [15]. Samples were measured using a Waters HPLC system (Waters, 717 autosampler, Waters PDA detector, and Waters 510 pump), Column (Waters Sunfire C18 3.5 *μ*m, 4.6 × 100 mm), and guard column (Waters Sentry Guard holder, WAT046910, Waters column joining tube assay WAT084080, Waters Sunfire 3.5 *μ*m 4.6 × 20 mm guard column, Part number 186002682) under the following conditions: flow rate 1.5 mL·min^−1^ and pressure 1000–2000 psi. Plasma *α*-tocopherol and *γ*-tocopherol were read at an absorbance of 240 nm, retinol was read at 420 nm, and lycopene, *α* carotene, and *β* carotene were read at 550 nm. Intra- and interassay CV were both <5%.

### 2.7. Statistical Analysis

Sample size was determined using a prospective power calculation performed for the main marker of interest (endothelial function, FMD) and this takes into consideration a 30% dropout rate. This was based on methods outlined by Altman [16].

Statistical analysis was completed using Microsoft Excel version 2010 (Microsoft, USA) and SPSS social statistics package version 19 (IBM, UK). One-sample Shapiro Wilks *W* tests were used to assess the distribution of the data. Parametric data were analysed using a repeated measures two-way analysis of variance (ANOVA), for two within participants' factors: time and exercise intensity trial. A Bonferroni-corrected paired samples *t*-test was used to further assess a significant interaction effect within participants. Nonparametric data was analysed using a number of Friedman Tests and post hoc analysis was completed using Bonferroni-corrected Wilcoxon Signed-Rank Tests. Statistical significance was accepted at a level of *P* < 0.05.

## 3. Results

### 3.1. Endothelial Function

#### 3.1.1. FMD Characteristics

**Figure 1:**
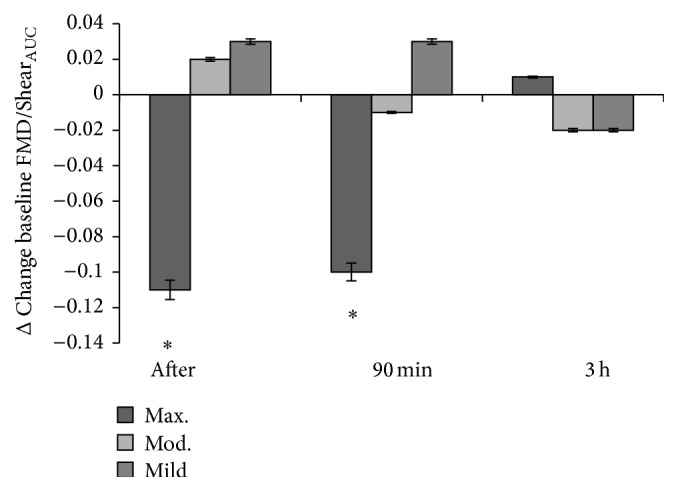
ΔFMD/Shear_AUC_ from baseline throughout the postexercise period. ^*∗*^ *P* ≤ 0.05 versus 3-hour postexercise maximal intensity trial. ΔFMD/Shear_AUC_ from baseline appears to decrease immediately after exercise and 90 minutes after exercise, respectively (*P* ≤ 0.05), before normalising at 3 hours after exercise.

No changes in baseline diameter, peak diameter, absolute change in diameter, or FMD were observed following any of the three exercise trials (*P* > 0.05; see Table 1), respectively. Nonetheless, within group differences were detected for ΔFMD/Shear_AUC_ change from baseline between immediately postexercise maximal trial versus 3-hour postexercise maximal trial (*P* = 0.014) and 90-minute postexercise maximal trial versus 3-hour postexercise maximal trial (*P* = 0.010; see Figure 1), respectively. Time to dilation was longer immediately following the maximal trial compared to baseline (*P* = 0.001). No changes in time to dilation were observed in any other exercise trial (*P* > 0.05). Shear rate was higher immediately after exercise in the maximal trial compared to values for baseline and 3 hours after exercise in the moderate trial (*time *×* trial interaction P* = 0.001 and *P* = 0.012, resp.) and after exercise and 90 minutes after exercise in the mild trial (*time *×* trial interaction* *P* = 0.006 and *P* = 0.005, resp.). Within group differences for shear rate were also detected in the maximal trial immediately after exercise compared with baseline and 3 hours after exercise (*P* < 0.001, resp.). See Table 1.

#### 3.1.2. ET-1

**Figure 2:**
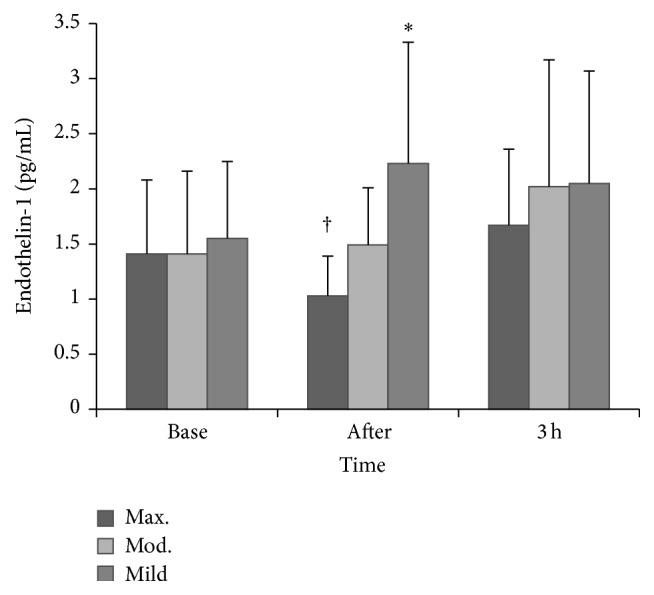
ET-1 prior to and following exercise. ^*∗*^ *P* ≤ 0.05 versus postmaximal exercise; ^†^ *P* ≤ 0.05 versus 3-hour postmaximal exercise.

ET-1 was higher immediately after exercise and 3 hours after exercise in the mild trial compared to the same points in the maximal trial (*time *×* trial interaction*, *P* = 0.008; *P* = 0.035), respectively. Compared to baseline, no change in ET-1 was observed immediately following the maximal trial (*P* = 0.096), but within group differences were observed between postexercise and 3 hours after exercise for ET-1 whereby ET-1 appeared to increase for this period (*P* = 0.002). No change in ET-1 was observed following moderate exercise (*P* > 0.05). See Figure 2.

### 3.2. Oxidative Stress Indices

**Figure 3:**
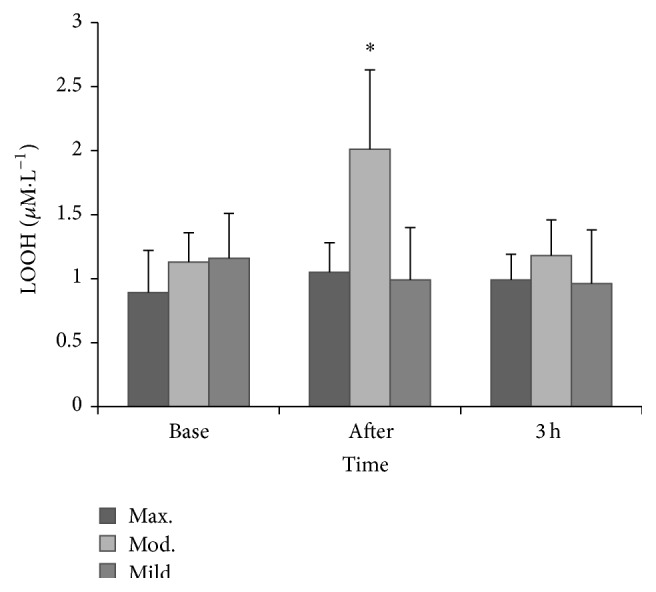
LOOH prior to and following exercise. ^*∗*^ *P* ≤ 0.001 versus all other time points.

Differences in LOOHs were observed between immediately post exercise in the moderate trial and the same time points in the other two trials (*time × trial interaction*, *P* < 0.001), respectively. Compared to baseline, LOOH increased immediately after exercise following moderate exercise (*P* < 0.001). This elevation in LOOHs decreased 3 hours after exercise (*P* < 0.001). There were no within group changes in LOOH following the mild and maximal exercise trials (*P* > 0.05, resp.). Data are presented in Figure 3.

**Figure 4:**
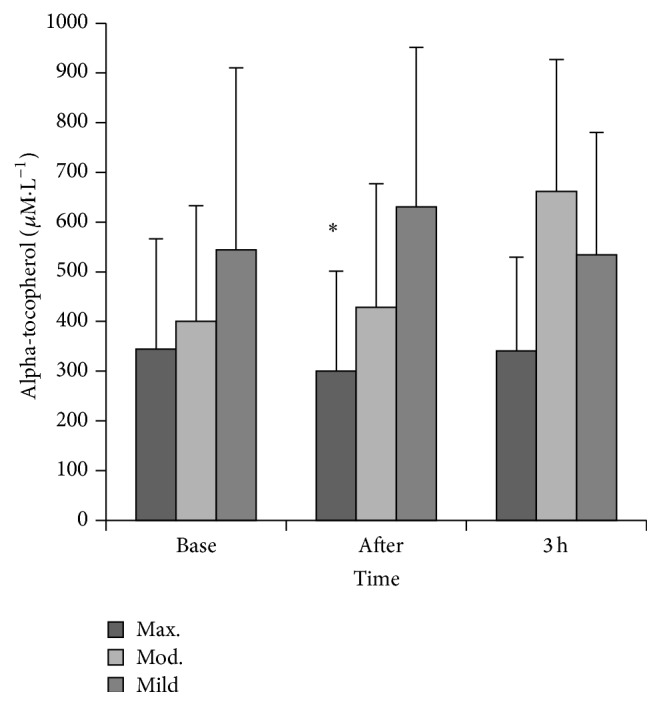
Alpha-tocopherol prior to and following exercise. ^*∗*^ *P* ≤ 0.05 versus postmoderate and postmild exercise.

No changes in lipid soluble antioxidants (gamma-tocopherol, lycopene, alpha-carotene, or beta carotene) were observed following any of the three exercise trials. Alpha-tocopherol was lower immediately after 5 minutes of exercise at 100%  $\dot{V}O_{2max}$ compared to immediately after 30 minutes of exercise at 55%  $\dot{V}O_{2max}$ (*χ* ^2^ = 10.43, *P* = 0.01; see Figure 4). The Friedman Test identified a difference in retinol at baseline between the exercise trials (*χ* ^2^ = 7.71, *P* = 0.02). However, Bonferroni-corrected and the post hoc Wilcoxon Signed-Rank Test did not identify significant changes in baseline values.

## 4. Discussion

The main findings of this investigation indicate that acute exercise of varying intensities does not elicit any detrimental effects to endothelial function (as characterised by FMD) throughout the postexercise period. Markers of lipid peroxidation (LOOH) were elevated immediately after exercise in the moderate trial only, but this change may be important in ROS-mediated exercise adaptations often associated with repeated bouts or exercise training [9] as no impairment in vessel function was evident. However, we provide tentative evidence that acute exercise may transiently affect the vascular response (ΔFMD/Shear_AUC_ from baseline) immediately after and 90 minutes after maximal exercise when compared to exercise at 55% and 75%  $\dot{V}O_{2max}$, respectively. Such differences may be due to a combination of factors including an increase in shear stress and a lower concentration of the vasoconstrictor, ET-1. Yet, this forms part of a transient vascular response for maximal exercise at 100%  $\dot{V}O_{2max}$ as ΔFMD/Shear_AUC_ from baseline was negative immediately after exercise in the maximal trial before normalising at 3 hours after exercise.

In the current study, no changes in FMD% were detected when exercising in the maximal trial compared to the other exercise intensities throughout the postexercise period. Although this clearly provides support for those advocating high/maximal intensity exercise, one confounder may be that the basal conditions differed at the various time points when FMD was determined. For instance, the baseline artery diameter for the maximal trial (presented in Table 1) is approximately 7% larger post-exercise than the baseline artery diameter recorded before exercise (baseline time-point). Since FMD is calculated as % change from baseline diameter, although the mean difference in baseline diameters was not statistically different, this individual difference in baseline diameter may be of importance. Thus, a larger baseline diameter, that is, postexercise, may mask a larger FMD. Alternatively, although a prospective power calculation was used, a large coefficient of variation for FMD might have been missed in the power analysis and a larger sample size may have benefit in future investigations to control for this observation. Significant interactions were observed for shear rate throughout the postexercise period (particularly in the maximal trial) and these observations suggest that increased shear stress following exercise could influence not only baseline diameter, but FMD as well. The lack of change in FMD may then be because the artery is preconditioned during the exercise and is closer to dilatory capacity, thereby decreasing the cuff-induced postischaemic shear stress [3]. Moreover, it has been recommended that FMD should be normalised by dividing the percentage of FMD by shear rate (AUC; FMD/Shear) to account for the large intersubject variability in reactive hyperaemia-induced shear stress [11, 12]. Following normalisation for FMD recommendations, we did indeed observe a relationship between FMD and shear at baseline and following acute exercise (data not shown). Although these data are in contrast with a previous report [17], disparity in our findings could be due to the intensity of our exercise. Clear trends appear to suggest that FMD/Shear_AUC_ was higher in the postexercise period for the maximal trial. However, when postexercise FMD/Shear_AUC_ was analysed in relation to the difference between baselines for each respective trial (ΔFMD/Shear_AUC_ from baseline), within group differences were observed in the maximal trial only. This observation may suggest (i) decreased vessel function in the period immediately following exercise and up until 90 minutes after exercise period compared to baseline and (ii) a restoration of vessel function at 3 hours after exercise compared to baseline. Accordingly, a biphasic response of FMD following acute exercise has been proposed whereby FMD appears to drop immediately following exercise, but then it normalises after approximately 1 hour [3, 18] although the normalisation process appears to have taken longer in the current study. The nature of this response seems to be influenced by the exercise mode, intensity, duration, and the timing of postexercise measurements [19]. The length of the high intensity bout may be an important predictor in the vascular response; reductions have been observed following longer bouts of high intensity exercise (>twenty minutes) whereas improvements have been noted in shorter bouts (<twenty minutes) [3]; clearly more work is needed to understand the biological value and drivers of this response.

Few studies have sought to measure endothelial function and oxidative stress throughout the postexercise period. Research suggests that an elevation in exercise intensity is accompanied by a corresponding increase in ROS which has the potential to decrease NO bioavailability. No changes in LOOHs were reported following maximal or mild exercise throughout the postexercise period, but lipid peroxidation increased immediately following the moderate exercise trial. Bloomer et al. [20] have demonstrated an increase in oxidative stress following 30 and 60 minutes of aerobic exercise at 70%  $\dot{V}O_{2peak}$, but, to our knowledge, no studies have reported a postexercise change in LOOHs following moderate exercise when the exercise bout is twenty minutes or less. A threshold intensity for elevating postexercise oxidative stress has been postulated to occur between 50 and 70%  $\dot{V}O_{2peak}$ when running between 20 and 60 minutes in trained subjects [7], and the current data suggests that exercise at the higher end of this range is more likely to elevate LOOHs. The functional and indeed quantitative significance of exercise-induced radical formation remains a source of contention, but it is likely that radical production during exercise may act as a signal to regulate molecular events such as the upregulation of antioxidant enzymes and heat shock proteins, events that are important in adaptation to exercise [21]. In fact, acute exercise-induced improvements in endothelial function have been recently attributed to the relaxation effect of some ROS [22]. The lack of change in LOOHs following the maximal trial may seem somewhat surprising, but one possible explanation could be that the concentration of LOOH production in this bout was not sufficient to overwhelm antioxidant capacity and therefore ROS clearance occurred at a sufficient rate to preserve NO bioavailability and not affect vascular function. Like FMD, the duration of the high intensity bout (5 minutes in the current study) may be an important modulator of the ROS response following exercise. A drop in alpha-tocopherol was observed in the maximal trial and it is plausible that these lipophilic antioxidants scavenged any exercise-induced ROS and prevented changes in LOOHs. Previous research has documented the protective effects of alpha-tocopherol on exercise-induced oxidative damage, particularly lipid peroxidation, and reductions of this chain-breaking antioxidant have been reported following acute treadmill exercise [23]. Other work has shown an increased intramuscular content of alpha-tocopherol following exercise possibly brought about by either a selective mobilization of alpha-tocopherol to counter any exercise-induced oxidative stress or an increase in lipoprotein delivery for hydrolysis by increased muscular blood flow during exercise [24].

Serum concentrations of the vasoactive substance ET-1 were found to be significantly lower immediately following the maximal bout when compared to mild exercise. To our knowledge, this is the first study to reveal such findings. Previous research indicates that aerobic exercise training can reduce ET-1. Yet, limited work exists assessing the acute response to exercise, but increases have been reported following a 30-minute high intensity bout [5, 25, 26]. The decreased ET-1 may be due to a greater uptake by ET-B receptors following maximal intensity exercise and may, to some extent, explain the favourable trends in FMD observed given that ET-1 is a potent vasoconstrictor [27]. Increased shear stress is a known stimulus for NO production and it is possible that the increased shear stress following maximal exercise induced a reduction of ET-1 release as NO is known to attenuate the production of ET-1, possibly via inhibiting superoxide [28, 29], but further work is required to explore this pathway. Alternatively, an increase in other vasoactive mediators or an exercise-induced elevation of circulating catecholamines could explain the comparative increase in ET-1 immediately following mild exercise.

Much of the current evidence highlighting the beneficial effects of high or maximal intensity exercise involves sprint interval training comprising four to six maximal sprints separated by periods of active recovery (typically three to five minutes). As such, one session may require 25–30 minutes of activity which falls within conventional physical activity guidelines. When matched for work, high intensity training can show similar benefits to traditional training, but less is known regarding the effects of low volume exercise [30], specifically when acutely examined. Therefore, in the present investigation, we sought to specifically examine the effects of a single bout of maximal exercise (five minutes in duration) in comparison to exercise bouts within the scope of the existing physical activity guidelines and found no detrimental effects to FMD and oxidative stress. We did observe transient reductions in ΔFMD/Shear_AUC_ from baseline, but these were restored at 3 hours after exercise and might be explained by a preconditioning effect of the maximal exercise. As with all studies, this investigation comes with some limitations. The three exercise bouts in the investigation were not matched for volume of work completed and thus it could be argued that any differences could be due to variances in the total work as opposed to the intensity per se and research is clearly needed to breach the gap. We believe the general public may not have the means to match exercise sessions for volume and, given that many individuals cite a lack of time as a perceived barrier to exercise, interventions that are time effective, but still yield beneficial health and fitness effects, are still of great interest to exercise physiologists. The collection of blood samples at all data points, commensurate with FMD measurements, may have also provided additional insights, but unfortunately these were not obtained in the current study. Future work should consider a longer follow-up within the postexercise period and larger sample sizes, which may help to reduce the variation evident in some of our data, for example, FMD. Finally, as the exercise bouts in this study were conducted following an overnight fast, control or standardizing of meal intake may also be considered for future research given the possible challenges of undertaking maximal intensity continuous running in the fasted state. However, no side-effects were reported during this trial.

## 5. Conclusions

Whilst the benefits of exercise for the individual and public health are widely recognised, less certainty is known about the precise mode, type, and duration of exercise required to attain such benefits. In recent years, the merit of low volume, high intensity exercise training has become increasingly prominent with data showing comparable benefits following high intensity interval training and traditional endurance-based training in skeletal muscle metabolic control and cardiovascular system function [30, 31]. Data from the current study seems to broadly support this theme by suggesting comparable effects of short duration maximal intensity exercise with mild and moderate exercise on measures of endothelial function (FMD) and oxidative stress. It is likely that exercise exerts changes to endothelial function via a multimechanistic pathway involving both physical and chemical stimuli; key mechanisms that may evoke changes to the vascular milieu include an increase in shear stress, a reduction in ET-1 concentration, and an alteration in oxidative stress. Further parallel research should be conducted to investigate the effect of low volume, high intensity exercise training on endothelial function in different population groups, specifically those at risk for cardiometabolic disorders, as this may represent a convenient alternative to traditional exercise regimes. Our findings provide tentative evidence that a single bout of maximal exercise may be a more time efficient approach than traditional exercise based on physical activity guidelines and this may have important implications for future public health recommendations.

## Figures and Tables

**Table 1:** Endothelial function data.

Trial/time	Baseline diameter (mm)	Peak diameter (mm)	Time to peak (s)	FMD (%)	Shear rate (AUC)	FMD : Shear_AUC_
Maximal						
Baseline	3.48 ± 0.45	3.68 ± 0.44	45.62^*∗*^ ± 15.14	5.40 ± 3.45	30780.22^*∗*^ ± 11458.38	0.20 ± 0.16
After	3.72 ± 0.42	3.94 ± 0.43	74.04 ± 21.93	6.12 ± 2.55	54147.79 ± 16042.47	0.12 ± 0.04
90 min	3.65 ± 0.41	3.58 ± 1.11 3.83 ± 0.43	62.46 ± 23.78	4.95 ± 3.62 6.3 ± 3.5	41063.70 ± 13477.47	0.13 ± 0.09
3 hours	3.62 ± 0.42	3.83 ± 0.43	44.92 ± 17.76	6.30 ± 3.50	30812.45^*∗*^ ± 10944.68	0.21 ± 0.15
Moderate						
Baseline	3.59 ± 0.50	3.71 ± 0.51	51.72 ± 25.29	3.66 ± 2.86	33929.82^*∗*^ ± 11250.44	0.12 ± 0.08
After	3.76 ± 0.53	3.98 ± 0.53	54.36 ± 21.40	6.08 ± 4.07	44216.42 ± 14675.47	0.14 ± 0.10
90 min	3.70 ± 0.51	3.87 ± 0.49	52.19 ± 20.29	4.73 ± 2.71	41062.87 ± 16845.44	0.12 ± 0.08
3 hours	3.70 ± 0.57	3.81 ± 0.55	64.06 ± 30.37	3.51 ± 3.21	35954.24 ± 8787.05	0.11 ± 0.10
Mild						
Baseline	3.56 ± 0.54	3.70 ± 0.47	54.78 ± 29.80	4.16 ± 4.13	37436.58 ± 13538.84	0.14 ± 0.16
After	3.68 ± 0.52	3.83 ± 0.50	56.56 ± 30.00	4.28 ± 3.77	36693.52 ± 15878.06	0.14 ± 0.12
90 min	3.69 ± 0.51	3.86 ± 0.48	49.69 ± 30.37	5.06 ± 4.57	34996.60 ± 11689.06	0.14 ± 0.16
3 hours	3.65 ± 0.50	3.74 ± 0.48	55.34 ± 23.47	3.51 ± 3.36	38935.07 ± 12666.07	0.10 ± 0.12

^*∗*^ *P* ≤ 0.05 versus postmaximal exercise.

## Abbreviations

FMD: Flow-mediated dilation
ET-1: Endothelin-1
ROS: Reactive oxygen species
NO: Nitric oxide
BMI: Body mass index
SST: Serum separating clot activator tubes
LOOH: Lipid hydroperoxides
HPLC: High pressure liquid chromatography
FOX: Ferrous iron/xylenol orange
ANOVA: Analysis of variance
HSD: Honestly significant test.
